# Effects of Protective American Football Headgear on Peripheral Vision Reaction Time and Visual Target Detection in Division I NCAA Football Players

**DOI:** 10.3390/sports7090213

**Published:** 2019-09-16

**Authors:** Rachel A. Miller, Rebecca R. Rogers, Tyler D. Williams, Mallory R. Marshall, Justin R. Moody, Robert W. Hensarling, Christopher G. Ballmann

**Affiliations:** Department of Kinesiology, Samford University, Birmingham, AL 35229, USA; rachrach977@gmail.com (R.A.M.); rrogers1@samford.edu (R.R.R.); twilli11@samford.edu (T.D.W.); mmarshal@samford.edu (M.R.M.); jrmoody@samford.edu (J.R.M.); rwhensar@samford.edu (R.W.H.)

**Keywords:** college football, dynavision, football helmet, eye shield, visor, facemask

## Abstract

The purpose of this study was to examine the effects of protective football headgear on peripheral vision reaction time and visual target detection. Twenty-five Division I NCAA football players (age = 20.5 yrs ± 0.9, height = 185.9 cm ± 6.8, body mass = 99.2 kg ± 19.2, BMI = 29.6 ± 4.5) participated. In a crossover counterbalanced study design, subjects participated in one visit with three conditions: Baseline (BL) without headgear, helmet only (HO), helmet with an eye shield (HE). Subjects completed a 1-min peripheral vision reaction time test for each condition separated by 3-min recovery periods. Tests were administered using a 64 light Dynavision D2 Visuomotor board. Target detection (total hit score) was higher during BL than HO (p < 0.001) and HE (p < 0.001). Average (p < 0.001), peak (p < 0.001), minimum (p < 0.001), and median (p < 0.001) peripheral reaction times were faster during BL than HO and HE. No significant differences were observed for any measures between HO and HE conditions (p > 0.05). Findings indicate that protective football headgear impaired reaction time to peripheral visual stimuli. The addition of an eye shield to the helmet had a small non-significant effect on reaction time and target detection. These results may hold important implications in helmet design and player safety.

## 1. Introduction

The first documented piece of protective American football headgear was developed in 1896 consisting of straps and earpieces with the intention of protecting the ears [1]. As tackling rules and regulations changed, wearing protective football headgear, specifically the helmet, became mandatory in the early mid 1900s in an effort to prevent brain and head injuries [2]. Over time, the football helmet has evolved from soft leather exteriors with no face protection to hard polycarbonate exteriors equipped with heavy gauge metal facemasks. While the earliest models of helmets allowed for clearer sight, the development of bulkier football headgear has led to more obstructions and visual impairment while wearing helmets [3]. Recently, additional protective headgear has been recommended in the form of eye shields in an effort to mitigate eye injuries [4]. Since unobstructed vision is necessary for players to perform sports-specific skills safely, knowledge of how modern football headgear affects the ability to respond to visual stimuli may be important to player safety and equipment design.

Most investigations of protective football headgear have focused on pthe revention of brain and head injuries and have shown equivocal efficacy [5,6,7,8]. Rowson et al. showed that while football helmets did not fully eliminate concussions, improved helmet design significantly decreased the rate of incidence in Division I National Collegiate Athletic Association (NCAA) football players [5]. Further supporting this, Collins et al. showed that newer helmet technology reduced concussion incidence over a three year period in high school football players [6]. However, multiple studies have refuted those findings and reported that evidence supporting the efficacy of football helmets in diminishing concussion is still unclear, possibly due to poor study design and control [7,8,9]. The use of face shields has been widely accepted to aid in reducing eye injury rates [4]. However, evidence suggests that face shields may not have a large impact concussion risk [7]. While the effect protective football headgear has on brain and head injuries has been widely studied, how it influences player ability to respond to peripheral visual stimuli is unknown. Given the fact that players can sustain up to ~1500 head impacts over the course of a season [10], being able to react quickly and appropriately may influence player safety, as previous evidence has shown that improved reaction time through training may decrease concussion incidence in football players [11].

The effect protective headgear has on peripheral vision and ability to respond to stimuli has largely been studied in sports and settings other than American football [12,13,14,15]. McKnight et al. reported that motorcyclists wearing a protective helmet had reduced peripheral vision compared to not wearing a helmet and this caused motorcyclists to compensate by increasing head rotation [12]. Ruedl et al. showed that wearing a ski helmet alone did not impair peripheral vision reaction time but wearing a ski helmet with protective eye goggles significantly increased reaction time [13]. Supporting the idea that protective eye equipment may impair reaction time, Dawson et al. revealed that wearing protective eyewear impaired peripheral reaction time in the horizontal field of vision [15]. Additionally, Kauffman et al. reported that athletes wearing sports goggles had impaired peripheral visual target detection during a peripheral vision performance test [16]. Given that protective headgear equipment may negatively impact visual field and reaction time in various sports and settings outside of American football, more research is needed to investigate how protective headgear unique to football influences visuomotor abilities.

A previous investigation using outdated technology reported that wearing protective football headgear obscured vision [3]. Other sports implementing newer and bulkier protective headgear technology have reported a decreased visual field [17,18]. Currently, it is unclear how modern football headgear technology impacts peripheral vision and the ability to respond to visual stimuli. While previous investigations have shown that protective eyewear impaired peripheral vision reaction time in other sports, it is currently unknown how protective eyewear specific to American football affects reaction time. Thus, investigation of how the latest protective football headgear technology affects the ability to respond to peripheral visual stimuli is warranted. The purpose of this study was to investigate the effects of wearing a football helmet equipped with and without an eye shield on peripheral vision reaction time and visual target detection. We hypothesized that wearing a football helmet only (HO) would significantly impair the ability to respond to visual stimuli in the periphery and that the addition of an eye shield to the helmet (HE) would exacerbate the impairment.

## 2. Materials and Methods

### 2.1. Subjects

Twenty five Male Division I NCAA football players (age = 20.5 yrs ± 0.9, height = 185.9 cm ± 6.8, body mass = 99.2 kg ± 19.2, BMI = 29.6 ± 4.5) were recruited to participate in this study. All subjects were currently active on a Division I NCAA football roster. Offensive (n = 12), defensive (n = 11), and special teams (n = 2) players participated. A health history questionnaire and a physical activity readiness questionnaire were administered to determine suitability for participation. All subjects underwent a vision-screening test by a certified athletic trainer using a Snellen eye chart to ensure normal visual acuity. Subjects were excluded if they had been diagnosed with a concussion in the past 6 months or reported any peripheral vision abnormalities. All subjects were asked to refrain from consuming alcohol, nicotine, or caffeine 12 h prior to participation [19]. Written informed consent was obtained from each subject prior to participation. The study was conducted in accordance with the Declaration of Helsinki and all procedures were approved by the Samford University Institutional Review Board (IRB).

### 2.2. Experimental Procedures

**Figure 1:**
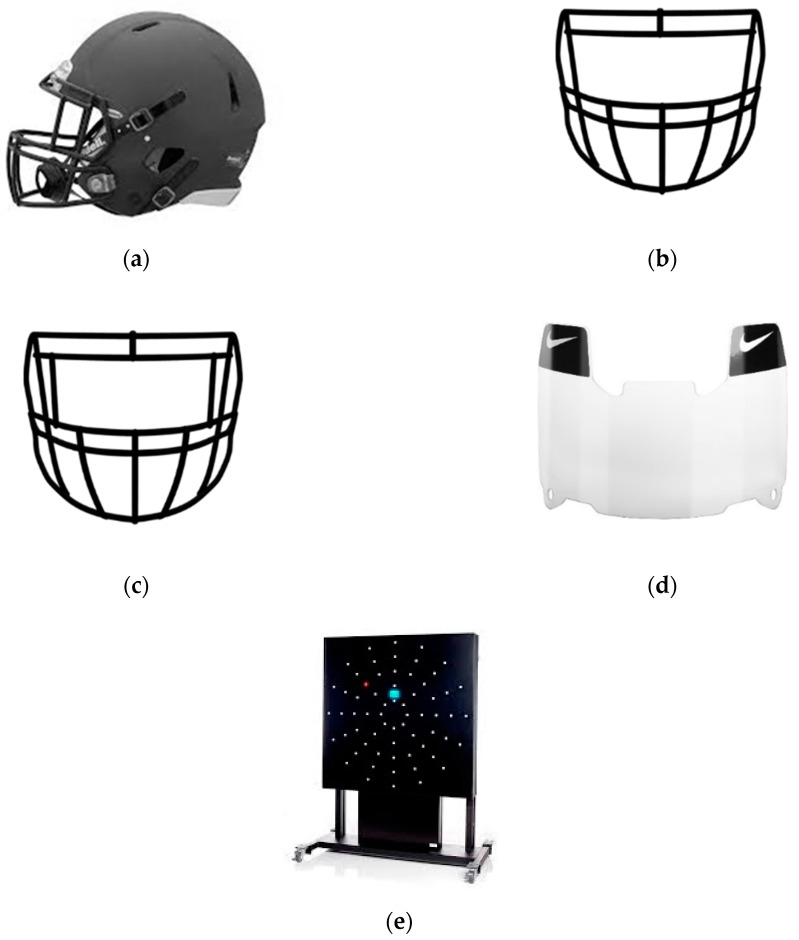
Dynavision and Football Headgear Equipment: (**a**) Riddell Speed Icon Helmet, (**b**) Riddell S2BD-SP facemask. (**c**) Riddell S2EGII-SP facemask. (**d**) Nike Gridiron Eye shield 2.0. (**e**) Dynavision D2 Visuomotor Board.

In a crossover counterbalanced study design, subjects participated in one visit and in a randomized order completed three different conditions: (1) Baseline (BL) with no headgear, (2) Helmet only (HO), and (3) Helmet with an eye shield (HE). Protective headgear is shown in Figure 1. Each subject was personally fitted with a Riddell Speed Icon helmet Figure 1A (Riddell, Elyria, OH, USA) by a certified athletic trainer. For helmet sizes small, medium, and large, a Riddell S2BD-SP (Figure 1B) (Riddell, Elyria, OH, USA) facemask was used. For the extra-large sized helmet, a Riddell S2EGII-SP (Figure 1C) (Riddell, Elyria, OH, USA) facemask was used. The facemasks were identical, with the exception of an extra bar on the S2EGII-SP facemask necessary due compatibility. For the HE condition, a clear Nike Gridiron Eye shield 2.0 (Figure 1D) (Nike, Beaverton, OR, USA) was attached to the facemask. The same helmet and facemask were used for both headgear conditions.

For experimental tests, subjects performed a single one-minute peripheral reaction time test for each condition for a total of three tests. All reaction time tests were administered on a Dynavision D2 Visuomotor board (Axtion Technology, Palatine, IL, USA) (Figure 1e). The D2 is a height adjustable board with 64 individual lighted buttons positioned in five concentric rings and four quadrants and has been reported by multiple groups to be reliable and valid for assessing reaction time [20,21]. Before the commencement of the tests, the Dynavision board was adjusted for each subject’s height to where the tachistoscope was at eye level. Standing position was determined for each participant to where they could comfortably reach the highest button on the outer fifth ring. Feet position was situated shoulder width apart. Feet and standing position were measured, marked, and documented to ensure identical position for all conditions. Prior to commencement of each test, participants were instructed to keep their eyes forward, focusing on the tachistoscope, and to hit each illuminated light as quickly as possible. The peripheral vision reaction time testing protocol was set up with the following parameters: one minute in length, proactive A mode, tachistoscope off, and only rings 3, 4, and 5 activated. Subjects were given a 3-min recovery period between each condition. The number of lights hit (total hit score) and the time until lights hit (peripheral reaction time) were recorded and analyzed for each condition.

### 2.3. Statistical Analysis

Outcomes were analyzed using SPSS 25 (IBM, Armonk, NY, USA). A 1 × 3 (Test × Condition) repeated measures ANOVA was used to statistically analyze all data and multiple comparisons with Bonfferoni correction were used to discern condition differences as warranted. Partial eta squared (η_p_^2^) was calculated for main effects. To detect magnitude of change in multiple comparisons, Cohen’s d effect sizes (d) were calculated using an effect size calculator with the following interpretations: 0.2—small; 0.5—moderate; 0.8—large [22]. Significance was set at p ≤ 0.05 a priori. All data are presented as mean ± standard deviation (SD).

## 3. Results

### 3.1. Visual Target Detection

**Figure 2:**
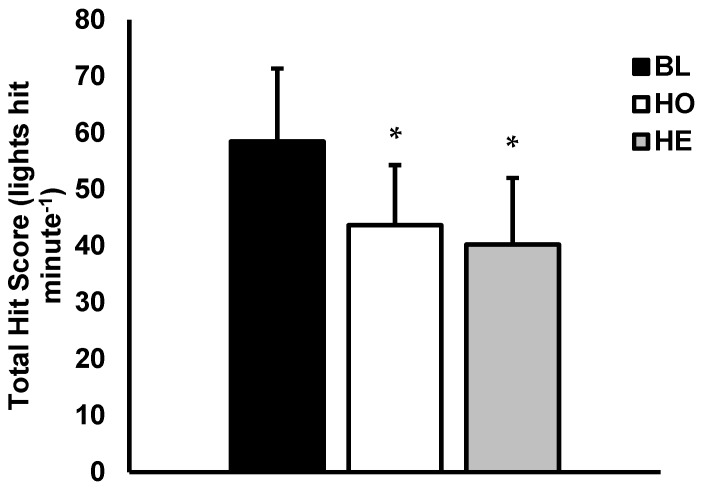
Visual field detection. Total Hit Score (lights hit · minute^−1^) for Baseline (BL), Helmet only (HO), and Helmet with eye shield (HE) conditions. Data are presented as mean ± SD. * Indicates significantly different from BL (p < 0.05).

Mean total hit score (lights hit · minute^−1^) is presented in Figure 2. Analysis revealed a main effect for condition (p < 0.001; η_p_^2^ = 0.74). Multiple comparisons showed t ttotal hit score was significantly higher during the BL versus HO condition and HE conditions (BL = 58 lights hit · minute^−1^ ± 12, HO = 43 lights hit · minute^−1^ ± 10, p < 0.001; d = 1.25; HE = 40 lights hit · minute^−1^ ± 11, p < 0.001; d = 1.47). No significant differences were found for mean total hit score between HO and HE (p = 0.226; d = 0.30).

### 3.2. Peripheral Reaction Time

The peripheral reaction times are presented in Table 1. For the average peripheral reaction time (sec), there was a main effect for condition (p < 0.001; η_p_^2^ = 0.62). Multiple comparisons showed average peripheral reaction time was significantly faster during the BL condition versus HO (p < 0.001; d = 1.22) and HE (p < 0.001; d = 1.13). There were no differences in average peripheral reaction times between HO and HE (p = 0.317; d = 0.37). For the peak peripheral reaction time (s), the fastest reaction time throughout the test, there was a main effect for condition (p < 0.001; η_p_^2^ = 0.56). The peak peripheral reaction time (s) was significantly faster during BL versus HO (p < 0.001; d = 0.77) and HE (p < 0.001; d = 0.78) conditions. No differences were observed for peak peripheral reaction time between HO and HE (p = 0.999; d = 0.03). An analysis revealed a main effect for condition (p < 0.001; η_p_^2^ = 0.53) for minimum peripheral reaction time (s), the slowest reaction time throughout the test. Multiple comparisons analyses revealed that minimum peripheral reaction time was significantly faster during BL versus HO (p < 0.001; d = 1.32) and HE (p < 0.001; d = 0.89). The slowest peripheral reaction time for HO and HE (p = 0.999; d = 0.14) did not differ. For the median peripheral reaction time (s), there was a main effect for condition (p < 0.001; η_p_^2^ = 0.71). The BL median peripheral reaction time was significantly faster than HO (p < 0.001; d = 1.01) and HE (p < 0.001; d = 1.25). The median peripheral reaction was not significantly different between HO and HE (p = 0.065; d = 0.39).

For ring 3 peripheral reaction time (s), there was a main effect for condition (p < 0.001; η_p_^2^ = 0.62). Ring 3 peripheral reaction time was significantly faster during BL versus HO (p < 0.001; d = 1.23) and HE (p = 0.011; d = 0.87) conditions. No differences were found between HO and HE (p = 0.999; d = 0.21) conditions. In ring 4, peripheral reaction time (s), analysis revealed a main effect for condition (p = 0.002; η_p_^2^ = 0.41). BL was significantly faster versus HO (p = 0.017; d = 0.70) and HE (0.002; d = 0.96). No differences were observed between HO and HE (p = 0.724; d = 0.26) conditions. Lastly, a main effect for condition (p < 0.001; η_p_^2^ = 0.62) was found for ring 5 peripheral reaction time (s). Multiple comparisons showed that BL was significantly faster versus both HO (p < 0.001; d = 1.25) and HE (p = 0.003; d = 0.98). No differences for peripheral reaction time in ring 5 were seen between HO and HE (p = 0.999; d = 0.19) conditions.

## 4. Discussion

Over time, protective football headgear has become bulkier, in efforts to prevent brain and head injuries [2,23]. Safety equipment such as the facemask and face shield have been adopted over time to prevent player injury. Previous evidence has shown that protective football headgear can obscure the visual field [3]. Being able to react to visual stimuli, especially in the periphery, may affect peak performance and player safety [24,25]. Thus, the purpose of the current study was to investigate the effects of wearing modern protective football headgear on peripheral vision reaction time and visual target detection. The findings reveal that wearing a helmet (HO) and a helmet with an eye shield (HE) decreased the total hit score during peripheral reaction time tests compared to baseline (BL). Furthermore, average, peak, minimum, median, ring 3, ring 4, and ring 5 reaction time were faster during BL compared to HO and HE. However, there were only small non-significant effects for all outcomes between HO and HE conditions. These findings may provide important information for football players, coaches, and practitioners concerning how protective headgear affects visuomotor ability.

Previous reports investigating protective football headgear, albeit outdated, reported an impaired visual field while wearing a football helmet and facemask [3]. Although the present study did not specifically map visual field impairments, our results are in agreement with these findings in that subjects were not able to respond to peripheral stimuli as fast while wearing football headgear. Indeed, previous evidence mapping peripheral vision impairments has shown a decreased ability to conduct motor tasks [26]. However, it is unclear what degree of peripheral vision impairment from football headgear is needed to cause a meaningful decrement in competition performance or football-specific tasks, leaving the need for follow up research. In contrast to this investigation, additional evidence from other sports such as skiing and cricket have shown that protective headgear had negligible or non-meaningful effects on peripheral vision and reaction time [13,17]. For example, Ruedl et al. showed that wearing a ski helmet did not impair peripheral reaction time during a compensatory-tracking-test [13]. The differences in findings may be because the ski helmet was not equipped with facial protection like the heavy gauge metal facemasks on American football helmets. This fact is bolstered by previous research showing that bulkier face protection impedes vision to a larger degree than more minimalist facial protection [3]. Thus, different models and types of modern football facemasks may differentially impair response to peripheral stimuli and should be the subject of subsequent analysis.

The present findings of small non-significant changes of peripheral vision impairment with an eye shield are in stark contrast with previous evidence in sports and settings other than football. Kauffman et al. reported that female athletes wearing sports goggles had impaired detection of peripheral visual stimuli [16]. Ruedl et al. showed that the addition of ski goggles to a helmet negatively impacted peripheral vision reaction time [13]. Disparities between these findings and the current findings may be due to eye protection design and distance between protective material and the eye. For example, ski and sports goggles have relatively thick frames that are very close to the orbital region of the face while football eye shields typically lack a frame and sit relatively further away from the eye. Since the thicker frame of ski and sports goggles are fixed so close to the eye, there may be a greater obstruction to peripheral vision. This is supported by previous evidence showing that hockey visors, which have no frame and sit relatively further away from the eye, similarly to football eye shields, have little to negligible influence on peripheral vision important for game-play [14]. However, how football eye shields affect specific vision angles that are important during competition or game-play is uncertain.

The results from this study showing a decreased ability to respond to peripheral visual stimuli while wearing protective football headgear may lead to practical applications to improve player safety. Clark et al. showed that providing football players vision training improved player safety [11]. Using a Dynavision training protocol, the same equipment used in the current investigation over a period of four seasons significantly decreased concussion incidence, missed playing time, and improved peripheral vision reaction time [11]. Despite these intriguing findings, all vision training was conducted without protective headgear, which may not fully translate to game-play and competition settings. Since football headgear impairs the ability to react to peripheral stimuli, vision training while wearing headgear may better replicate real-world settings and could potentially lead to more relevant visuomotor adaptations aiding in preventing injury. Supporting this, Brenton et al. showed that visual training with temporal occlusion improved sports-specific visuomotor skills in well-trained athletes [27]. However, it should be cautioned that the current study did not conduct vision training, leaving the need for longitudinal investigation on vision training while wearing protective football headgear.

While the current investigation presented novel information on how protective football headgear influences peripheral vision reaction time, there were several limitations. First, although almost identical, the same model of facemask was not used for every participant. This was due, in part, to the size of the helmet, which necessitated a slightly different facemask for compatibility. Thus, we cannot rule out the possibility that players wearing the extra-large sized helmet may have had peripheral vision affected differently. Furthermore, only one brand and model of helmet and eye shield were used. The current findings may not be generalizable to other brands and models available. Lastly, common disadvantages of eye shield use during competition such as fogging, condensation, and glare, were not incorporated into the study design. Therefore, we cannot rule out that these factors may influence peripheral vision and reaction time if present during game play. In conclusion, wearing protective football headgear impairs player ability to respond to peripheral stimuli. However, the addition of an eye shield to a helmet only caused a trivial effect of further impairment. Future research should examine other brands and models of protective football headgear and investigate whether vision training can improve reaction time outcomes in efforts to improve player safety.

## Figures and Tables

**Table 1:** Peripheral vision reaction times (s). Data are presented as mean ± SD. * indicates significantly different from BL (p < 0.05).

Reaction Time	Baseline (BL)	Helmet Only (HO)	Helmet + Eye Shield (HE)
Average (s)	1.08 ± 0.26	1.45 ± 0.35 *	1.68 ± 0.81 *
Peak (s)	0.57 ± 0.11	0.65 ± 0.11*	0.66 ± 0.12 *
Minimum (s)	3.07 ± 1.03	5.28 ± 2.29 *	5.83 ± 5.13 *
Median (s)	0.88 ± 0.17	1.08 ± 0.21 *	1.19 ± 0.32 *
Ring 3 (s)	0.84 ± 0.18	1.08 ± 0.21 *	1.16 ± 0.56 *
Ring 4 (s)	1.13 ± 0.28	1.37 ± 0.41 *	1.49 ± 0.46 *
Ring 5 (s)	1.24 ± 0.36	1.77 ± 0.50 *	1.92 ± 1.03 *
